# The Equivalency between Logic Petri Workflow Nets and Workflow Nets

**DOI:** 10.1155/2015/121492

**Published:** 2015-03-03

**Authors:** Jing Wang, ShuXia Yu, YuYue Du

**Affiliations:** Shandong University of Science and Technology, Qingdao, China

## Abstract

Logic Petri nets (LPNs) can describe and analyze batch processing functions and passing value indeterminacy in cooperative systems. Logic Petri workflow nets (LPWNs) are proposed based on LPNs in this paper. Process mining is regarded as an important bridge between modeling and analysis of data mining and business process. Workflow nets (WF-nets) are the extension to Petri nets (PNs), and have successfully been used to process mining. Some shortcomings cannot be avoided in process mining, such as duplicate tasks, invisible tasks, and the noise of logs. The online shop in electronic commerce in this paper is modeled to prove the equivalence between LPWNs and WF-nets, and advantages of LPWNs are presented.

## 1. Introduction

Petri nets (PNs) [1] are a process modeling technique applied to the simulation and analysis of distributed systems, and PNs are also an effective description and analysis tool for many fields. With the continuous development of PN theory and the increasing popularity of its application, some of their extensions have been defined, such as colored [2], time [3], fuzzy [4], and stochastic PNs [5]. Logic Petri nets [6–8] are the abstract and extension of high-level PNs and have been applied efficiently to the modeling and analysis of Web services, cooperative systems, and electronic commerce. Transitions restricted by logic expressions are called logic transitions. The inputs and outputs can be described by logic transitions in LPNs. Based on LPNs, the definition of LPWNs is proposed in this paper. An LPWN is logic Petri net with a dedicated source place where the process starts and a dedicated sink place where the process ends. Moreover, all nodes are on at least a path from source to sink.

Larger online shops produce a great quantity of transaction records every day. How to find valuable information in these records is a meaningful task. These records are called event logs in process mining, which are the starting point of process mining [9, 10]. When modeling business processes in terms of Petri nets, a subclass of Petri nets known as Workflow nets is considered [11–14]. WF-nets are also a natural representation for process mining. Process mining [15, 16] is a young cross field and crosses the computational intelligence and data mining field to the modeling process and analysis area. Process mining is regarded as an important bridge between modeling and analysis of data mining and business process [17–19]. LPWNs and WF-nets are evolutions of PNs. The LPWN will be introduced into process mining in our later work, so the equivalency between LPWNs and WF-nets is firstly proved by an online shop model in this paper. Compared with WF-nets, LPWNs can well describe and analyze batch processing functions and passing value indeterminacy in cooperative systems and effectively alleviate the state space explosion problem to an extent.

The rest of this paper is organized as follows.  Section 2 reviews definitions of PNs, WF-nets, and LPNs, and the standard forms of logic expressions and LPWNs are put forward. A simple LPWN model is given to explain how the LPWN works. In order to prove the equivalence between LPWNs and WF-nets, isomorphism and equivalent definitions are proposed in Section 3.  Theorem 8 has been proved on the basis of isomorphism and equivalent definitions, and the constructing algorithm of an equivalent WF-net from an LPWN is presented. In Section 4, Theorem 8 and the algorithm are illustrated by an online shop model. Concluding remarks are made in Section 4.

## 2. Logic Petri Workflow Nets

This section introduces some basic definitions about PNs, LPNs, and WF-nets.

**Definition 1 (see [8]).**

PN = (*P*, *T*; *F*, *M*) is a marked PN, where

(1) *N* = (*P*, *T*; *F*) is a net;
(2) *M* : *P* → ℕ is a marking function, where *M*_0_ is the initial marking and ℕ → {0,1, 2,…};
(3) transition firing rules are as follows:
  (a) *t* is enabled at *M* if for all *p* ∈ ^•^ *t* : *M*(*p*) = 1, represented by *M*[*t*>;
  (b) if *t* is enabled, it can fire, and a new marking *M*′ is generated from *M*, represented by *M*[*t* > *M*′, where
    (1) M′p=Mp+1,if  p∈t•−t•Mp−1,if  p∈t•−t•Mp,else.

**Definition 2 (see [8]).**

Let PN = (*P*, *T*; *F*, *M*_0_) be a Petri net and *t* ^#^ a fresh identifier not in *P* ∪ *T*. The PN is a workflow net (WF-net) if and only if

- *P* contains an input place *i* (also called source place) such that ^•^ *i* = *⌀*;
- *P* contains an output place *o* (also called sink place) such that *o* ^•^ = *⌀*;
- PN^#^ = (*P*, *T* ∪ {*t* ^#^}, *F* ∪ {(*o*, *t* ^#^), (*t* ^#^, *i*)}) is strongly connected.

There is a directed path between any pair of nodes in PN.

**Definition 3 (see [8]).**

LPN = (*P*, *T*; *F*, *I*, *O*, *M*) is a logic Petri net where

(1) *P* is a finite set of places;
(2) *T* = *T*_*D*_ ∪ *T*_*I*_ ∪ *T*_*O*_ is a finite set of transitions, *P* ∪ *T* ≠ *⌀*, *P*∩*T* = *⌀*, for all *t* ∈ *T*_*I*_ ∪ *T*_*O*_ : ^•^ *t*∩*t* ^•^ = *⌀*, where
  (a) *T*_*D*_ denotes a set of traditional transitions;
  (b) *T*_*I*_ denotes a set of logic input transitions, where, for all *t* ∈ *T*_*I*_, the input places of *t* are restricted by a logic input expression *f*_*I*_(*t*);
  (c) *T*_*O*_ denotes a set of logic output transitions, where, for all *t* ∈ *T*_*O*_, the output places of *t* are restricted by a logic output expression *f*_*O*_(*t*);
(3) *F*⊆(*P* × *T*)∪(*T* × *P*) is a finite set of directed arcs;
(4) *I* is a mapping from a logic input transition to a logic input expression; that is
  (2) $$\begin{array}{c} \forall t\in T_{I},I\left(t\right)\;f_{I}\left(t\right)=A_{1}\vee A_{2}\vee \cdots \vee A_{m}; \end{array}$$
(5) *O* is a mapping from a logic output transition to a logic input expression; that is
  (3) $$\begin{array}{c} \forall t\in T_{O},O\left(t\right)\;f_{O}\left(t\right)=B_{1}\vee B_{2}\vee \cdots \vee B_{n}; \end{array}$$
(6) *M* : *P* → {0, 1} is a marking function, where, for all *p* ∈ *P*, *M*(*p*) is the number of tokens in *p*;
(7) Transition firing rules are as follows:
  (a) for all *t* ∈ *T*_*D*_, the firing rules of *t* are the same as in PNs;
  (b) for all *t* ∈ *T*_*I*_, *t* is enabled only if ∃*A*_*i*_; make *f*_*I*_(*t*)|_*M*_ = *T*_•_ *T*_•_, *M*[*t* > *M*′, where, for all *p* ∈ ^•^ *t* and *p* ∈ *A*_*i*_, *M*′(*p*) = *M*(*p*) − 1; for all *p* ∈ ^•^ *t* and *p* ∉ *A*_*i*_, *M*′(*p*) = *M*(*p*); for all *p* ∈ *t* ^•^, *M*′(*p*) = *M*(*p*) + 1; and, for all *p* ∉ ^•^ *t* ∪ *t* ^•^, *M*′(*p*) = *M*(*p*);
  (c) for all *t* ∈ *T*_*O*_, *t* is enabled only if for all *p* ∈ ^•^ *t* : *M*(*p*) = 1. *M*[*t* > *M*′, where for all *p* ∈ ^•^ *t* : *M*′(*p*) = *M*(*p*) − 1; for all *p* ∉ ^•^ *t* ∪ *t* ^•^ : *M*′(*p*) = *M*(*p*); for all *p* ∈ *t* ^•^ and *p* ∈ *B*_*i*_ should satisfy *f*_*O*_(*t*)|_*M*′_ = *T*_•_ *T*_•_; and for all *p* ∈ *t* ^•^ and *p* ∉ *B*_*i*_, *M*′(*p*) = *M*(*p*).

LPNs are the abstract and extension of IPNs and high-level PNs. In Definition 3, a logic input/output transition is restricted by the logic input/output expression *f*_*I*_(*t*)/*f*_*O*_(*t*) in LPNs. All logic input/output transitions are called logic transitions. The logic expressions can describe the indeterminacy of values in input and output places. *A*_*i*_ and *B*_*i*_ represent input and output ways of logic transitions, respectively. They are not the disjunctive normal of *f*_*I*_(*t*)/*f*_*O*_(*t*).

**Definition 4 .**

Suppose that a logic input/output transition *t* is restricted by *f*_*I*_(*t*)/*f*_*O*_(*t*), and the standard form is as follows.

For a logic input transition *t*, the standard form of *f*_*I*_(*t*) = *A*_1_∨*A*_2_∨⋯∨*A*_*m*_ can be obtained by

(4) Ai=Ai,if  Ai=t•Ai∧¬p,if  Ai≠t•,  ∃p∈t  •.

For a logic input transition *t*, the standard form of *f*_*O*_(*t*) = *B*_1_∨*B*_2_∨⋯∨*B*_*n*_ can be obtained by

(5) $$\begin{array}{c} B_{i}=\left\{\begin{array}{cc} B_{i}, & \text{if}\;\left|B_{i}\right|=\left|t^{•}\right|\\ B_{i}\wedge \neg p, & \text{if}\;\left|B_{i}\right|\neq \left|t^{•}\right|,\;\;\exists p\in t^{•}. \end{array}\right. \end{array}$$

This definition puts forward the standard form of logic expression. *A*_*i*_ and *B*_*i*_ are called the standard minterms.

**Definition 5 .**

Let LPN = (*P*, *T*; *F*, *I*, *O*, *M*) be a logic Petri net, and the LPN is a logic Petri workflow net (LPWN) if and only if

- LPN has *P* = *P*_*C*_ ∪ *P*_*D*_, where *P*_*C*_/*P*_*D*_ are control/data place sets;
- there is a source place *i* ∈ *P*_*C*_ such that ^•^ *i* = *⌀*; there is a sink place *o* ∈ *P*_*C*_ such that *o* ^•^ = *⌀*;
- there is a directed path between the source place and sink place.

From Definitions 3 and 5, the LPWNs and WF-nets have the same kind of starting place with a token and an ending place.

**Figure 1:**
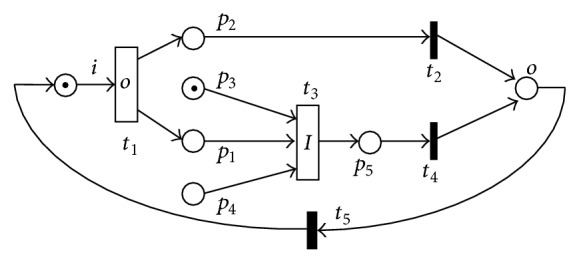
An LPWN model *N*_1_.

Figure 1 shows a simple LPWN model. *t*_2_, *t*_4_, and *t*_5_ are three traditional transitions. *t*_1_ restricted by *f*_*O*_ is a logic output transition; *f*_*O*_(*t*_1_) = *B*_1_∨*B*_2_, where *B*_1_ = *p*_1_, *B*_2_ = *p*_2_; *t*_3_ restricted by *f*_*I*_ is a logic input transition; *f*_*I*_(*t*_3_) = *A*_1_∨*A*_2_, where *A*_1_ = *p*_1_∧*p*_3_, *A*_2_ = *p*_1_∧*p*_4_.

From Definition 4, standard forms of *f*_*O*_(*t*_1_) and *f*_*I*_(*t*_3_) are *f*_*O*_(*t*_1_) = (*p*_1_∧¬*p*_2_)∨(¬*p*_1_∧*p*_2_) and *f*_*I*_(*t*_3_) = (*p*_1_∧¬*p*_3_∧*p*_4_)∨(*p*_1_∧*p*_3_∧¬*p*_4_), respectively. Note that each place of a logic expression has a logic value at marking *M* in an LPWN, and, by substituting the values of all places into the logic expression, the expression corresponds to a logic value.

In the LPWN model of Figure 1, *M* = (1, 0, 0, 1, 0, 0, 0), *O* = {*p*_1_, *p*_2_}, and *I* = {*p*_1_, *p*_3_, *p*_4_}. For the source place *i* having a token, the transition *t*_1_ can fire. Suppose that *t*_1_ fires, *p*_1_ has a token, and *M*[*t*_1_ > *M*′. From Definition 3, *M*′ = (0, 1, 0, 1, 0, 0, 0), having *p*_1_|_*M*′_ = *T*_•_ *T*_•_, *p*_3_|_*M*′_ = *T*_•_ *T*_•_, *p*_4_|_*M*′_ = *T*_•_ *F*_•_, and *f*_*I*_|_*M*′_ = (*T*_•_ *T*_•_∧*T*_•_ *F*_•_∧*T*_•_ *F*_•_)∨(*T*_•_ *T*_•_∧*T*_•_ *T*_•_∧*T*_•_ *T*_•_) = *T*_•_ *F*_•_∨*T*_•_ *T*_•_ = *T*_•_ *T*_•_. The logic expression *f*_*I*_ is satisfied, *t*_3_ is enabled, and *M*′[*t* > *M*′′. From condition (b) of Definition 3, *M*′′ = (0, 0, 0, 0, 0, 1, 0).

## 3. Transforming an LPWN into an Equivalent WF-Net

This section puts forward isomorphism and equivalent definitions to prove the equivalence between LPWNs and WF-nets.

**Definition 6 .**

Let Σ_1_ = (*P*, *T*; *F*, *I*, *O*, *M*) be an LPWN and Σ_2_ = (*P*′, *T*′; *F*′, *M*′) a WF-net. RG(Σ_*i*_) is the reachable tree of Σ_*i*_, and *R*(Σ_*i*_) is the node set of RG(Σ_*i*_),  *i* = 1,2. If there exists a bijective function *f* : *R*(Σ_1_) → *R*(Σ_2_), such that, for all *M*_1_, *M*_2_ ∈ *R*(Σ_1_), *t* ∈ *T*, *M*_1_[*t* > *M*_2_, ⇒∃*t*′ ∈ *T*′, *f*(*M*_1_)[*t*′ > *f*(*M*_2_). Then, RG(Σ_1_) and RG(Σ_2_) are isomorphic.

**Definition 7 .**

Let Σ_1_ = (*P*, *T*; *F*, *I*, *O*, *M*) be an LPWN and Σ_2_ = (*P*′, *T*′; *F*′, *I*′, *M*′) a WF-net. Σ_1_ and Σ_2_ are equivalent if and only if RG(Σ_1_) and RG(Σ_2_) are isomorphic.

Based on Definitions 6 and 7, a theorem is given.

**Theorem 8 .**

For any LPWN, there exists an equivalent WF-net.

**Proof**

Consider the following.

*Step 1.* Constructing an equivalent WF-net is as follows.

Let Σ_1_ = (*P*, *T*; *F*, *I*, *O*, *M*_0_) be an LPWN, and the deterministic WF-net Σ_2_ = (*P*′, *T*′; *F*′, *M*′) being equivalent to Σ_1_ should be constructed at the very start.

For all *t* ∈ *T*, there are three conditions to transform a transition of Σ_1_ into one or more corresponding transitions Σ_2_.

*Step 1.1*. For *t*_*i*_ ∈ *T*_*D*_, let *t*_*i*_ ∈ *T*′; for all *p* ∈ *P*, if (*p*, *t*_*i*_) ∈ *F*, then (*p*, *t*_*i*_) ∈ *F*′; and if (*t*_*i*_, *p*) ∈ *F*, then (*t*_*i*_, *p*) ∈ *F*′.

*Step 1.2*. For *t*_*i*_ ∈ *T*_*I*_, let ^•^ *t*_*i*_ = {*p*_*i*1_, *p*_*i*2_,…, *p*_*ik*_}; *f*_*I*_(*t*_*i*_) = *A*_*i*1_∨*A*_*i*2_∨⋯∨*A*_*im*_; *t*_*i*_ is restricted by the standard logic input expression *f*_*I*_(*t*_*i*_). There are *m* standard minterms of *f*_*I*_(*t*_*i*_), and each minterm corresponds to a transition of Σ_2_. That is, the logic input transition *t*_*i*_ in Σ_1_ can be represented equivalently by a set including *m* traditional transitions in Σ_2_. The set is constructed in detail as follows.

For any standard minterm *A*_*ij*_, where *j* ∈ {1,2,…, *m*}, assume that *A*_*ij*_ corresponds to the transition *t*_*ij*_ in Σ_2_; that is, *t*_*ij*_ ∈ *T*′. Then, the arc set related to *t*_*ij*_ is defined. For all *p*_*il*_ ∈ ^•^ *t*_*i*_, where *l* ∈ {1,2,…, *k*}, if *p*_*il*_ in *A*_*ij*_ is *T*_•_ *T*_•_, we have (*p*_*il*_, *t*_*ij*_) ∈ *F*′; for all *p* ∈ *t*_*i*_^•^, we have (*t*_*i*_, *p*_*il*_) ∈ *F*′, where *l* ∈ {1,2,…, *k*}.

*Step 1.3*. For *t*_*i*_ ∈ *T*_*O*_, let *t*_*i*_^•^ = {*p*_*i*1_, *p*_*i*2_,…, *p*_*ik*_}; *f*_*O*_(*t*_*i*_) = *B*_*i*1_∨*B*_*i*2_∨⋯∨*B*_*in*_; *t*_*i*_ is restricted by the standard logic output expression *f*_*O*_(*t*_*i*_). There are *n* standard minterms of *f*_*O*_(*t*_*i*_), and each minterm corresponds to a transition of Σ_2_. That is, the logic output transition *t*_*i*_ in Σ_1_ can be represented equivalently by a set including *n* traditional transitions in Σ_2_. The set is constructed in detail as follows.

For any standard minterm *B*_*ij*_, where *j* ∈ {1,2,…, *n*}, assume that *B*_*ij*_ corresponds to the transition *t*_*ij*_ in Σ_2_; that is, *t*_*ij*_ ∈ *T*′. Then, the arc set related to *t*_*ij*_ is defined. For all *p*_*il*_ ∈ *t*_*i*_^•^, where *l* ∈ {1,2,…, *k*}, if *p*_*il*_ in *B*_*ij*_ is *T*_•_ *T*_•_, we have (*t*_*ij*_, *p*_*il*_) ∈ *F*′; for all *p* ∈ ^•^ *t*_*i*_, we have (*p*, *t*_*ij*_) ∈ *F*′, where *j* ∈ {1,2,…, *n*}.

*Step 2.* Proof that the constructing WF-net Σ_2_ is equal to Σ_1_.

Based on Step 1, the place set *P* and the initial marking *M*_0_ in Σ_1_ are the same as those in Σ_2_; that is, *P* = *P*′, *M*_0_ = *M*′, but the transition set *T* and the flow set *F* are not; that is, *T* ≠ *T*′, *F* ≠ *F*′, and |*T*| ≤ |*T*′|, |*F*| ≤ |*F*′|, where |*T*| denotes the size of set *T*. Firing a transition of Σ_1_ corresponds to firing a transition of Σ_2_; that is, if a transition is enabled in Σ_1_, then there must be an enabled transition in Σ_2_ and it is unique. Since Σ_1_ and Σ_2_ have the same initial marking, the equivalence between Σ_1_ and Σ_2_ is proved on the basis of the reachable marking graph.

In Σ_1_, for all *M*_1_, *M*_2_ ∈ *R*(Σ_1_), *t*_*i*_ ∈ *T*; if *M*_1_[*t*_*i*_ > *M*_2_, then there is a mapping function *f* : *R*(Σ_1_) → *R*(Σ_2_) based on Step 1; we have *f*(*M*_1_) = *M*_1_ and *f*(*M*_2_) = *M*_2_. In Σ_2_, if *t*_*i*_ ∈ *T*_*D*_, then ∃*t*_*i*_′ ∈ *T*′: *t*_*i*_′ = *t*_*i*_ and *f*(*M*_1_)[*t*_*i*_′ > *f*(*M*_2_); if *t* ∈ *T*_*I*_ ∪ *T*_*O*_, then ∃*t*_*ij*_′ ∈ *T*′; we have *f*(*M*_1_)[*t*_*ij*_′ > *f*(*M*_2_).*f* is an identity mapping and satisfies injective and surjection requirements at *M* ∈ *R*(*M*_0_). That is, Σ_1_ and Σ_2_ have the same behavior characteristics. Moreover, the structure of Σ_2_ is unique since its standard form is only one. So *f* is a bijective function, and RG(Σ_1_) and RG(Σ_2_) are isomorphic. Based on Definition 7, Σ_1_ and Σ_2_ are equivalent.

Based on Theorem 8 and the construction of Σ_2_, the construction algorithm of an equivalent WF-net from an LPWN can be obtained.

In Algorithm 1, the equivalent WF-net has the same place set and traditional transitions compared with its corresponding LPWN. Their differences are the logic transitions and flows. Next, an example is used to prove the correctness and appropriateness of Theorem 8 and Algorithm 1.

## 4. A Case

**Figure 2:**
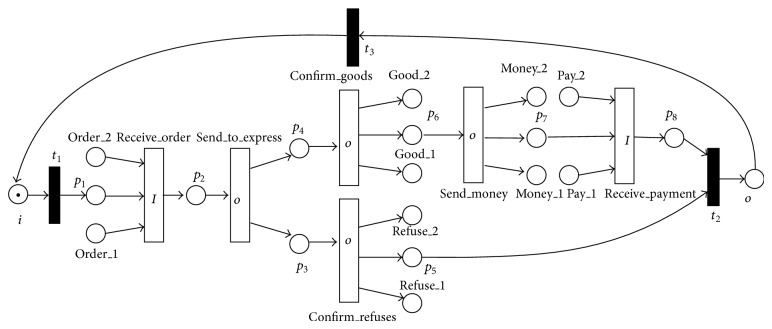
An online shop is modeled by the LPWN *N*_1_.

In this section, the work processes of an online shop in electronic commerce shown in Figure 2 are modeled by the LPWN, and the validity and usefulness of the presented method are illustrated based on the analysis of the model. Functions of the online shop are modeled by transitions. For example, the transition receive_order represents that the shop owner will get an order from the client, and it is limited by the logic expression *f*_*I*_(receive_order). Based on Definition 4, all logic transitions and their standard items are shown in Table 1.

Next, the LPWN *N*_1_ shown in Figure 2 will be transformed into its equivalent WF-net.

In Figure 2, the logic input transition receive_order can be transformed into three traditional transitions as follows.

The receive_order is a logic input transition restricted by *f*_*I*_(receive_order) = *A*_11_∨*A*_12_∨*A*_13_, where *A*_11_ = *p*_1_∧order_1∧¬order_2, *A*_12_ = *p*_1_∧order_1∧order_2, and *A*_13_ = *p*_1_∧¬order_1∧order_2. The receive_order has three ways to transform tokens. For example, (*p*_1_∧order_1∧¬order_2) represents *p*_1_ and order_1 loses a token and order_2 does not lose a token after the receive_order fires. From Algorithm 1, in the equivalent WF-net, the transition receive_order can be transformed into *ro*1, *ro*2, and *ro*3, and they are three traditional transitions. Flows (*p*_1_, receive_order), (order_1, receive_order), and (order_2, receive_order) are transformed into seven flows (*p*_1_, *ro*1), (*p*_1_, *ro*2), (*p*_1_, *ro*3), (order_1, *ro*1), (order_1, *ro*3), (order_2, *ro*2), and (order_2, *ro*3). The flow (receive_order, *p*_2_) is transformed into three flows (*ro*1, *p*_2_), (*ro*2, *p*_2_), and (*ro*3, *p*_2_). The input transition receive_payment can also be transformed by this method.

**Figure 3:**
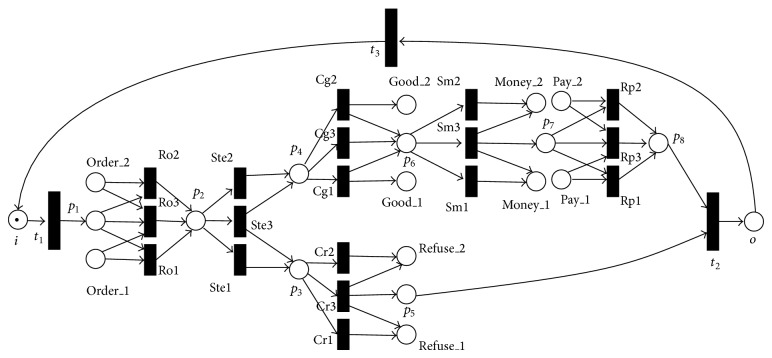
The LPWN *N*_1_ is transformed into its equivalent WF-net.

In Figure 2, the logic input transition send_to_express can be transformed into traditional transitions shown in Figure 3 as follows.

The send_to_express is a logic output transition restricted by *f*_*O*_(send_to_express) = *B*_11_∨*B*_12_∨*B*_13_, where *B*_11_ = *p*_3_∧¬*p*_4_, *B*_12_ = *p*_3_∧*p*_4_, and *B*_13_ = ¬*p*_3_∧*p*_4_. The send_to_express has three ways to transform tokens. For example, (*p*_3_∧¬*p*_4_) represents that *p*_3_ gets a token and *p*_4_ does not get a token after the send_to_express fires. From Algorithm 1, in the equivalent WF-net, the logic output transition send_to_express can be transformed into ste1, ste2, and ste3, and they are three traditional transitions. Flows (send_to_express, *p*_3_), (send_to_express, *p*_4_) are transformed into four flows (ste1, *p*_3_), (ste3, *p*_3_), (ste3, *p*_4_), and (ste2, *p*_4_). The flow (*p*_2_, send_to_express) is transformed into three flows (*p*_2_, ste1), (*p*_2_, ste2), and (*p*_2_, ste3). Other output transitions confirm_refuses, confirm_goods, and send_money can be transformed by this method.

In Figure 2, *t*_1_, *t*_2_, and *t*_3_ are three traditional transitions, and places, transitions, and flows related to them do not change. Based on the above method, the equivalent WF-net can be obtained in Figure 3.

From Figures 2 and 3, the WF-net consists of 21 transitions and 58 flows while its equivalent LPWN model has 9 transitions and 30 flows, and the number of their places is the same. The rates of transitions and flows descending from its WF-net to its LPWN in the example are 42.86% and 51.72%, respectively. There is a conclusion that LPWNs and WF-nets are equal to modeling, and LPWNs compared with WF-nets can alleviate the state space explosion problem.

## 5. Conclusions

Based on the definition of LPNs, LPWNs are proposed in this paper. An LPWN is logic Petri net with a dedicated source place where the process starts and a dedicated sink place where the process ends. Moreover, all nodes are on a path from source to sink.  Theorem 8 has been proved, and Algorithm 1 used to construct an equivalent WF-net from an LPWN is put forward. Effectiveness and practicality of the proposed algorithm have been exemplified by the online shop model.

In further work, the fundamental properties of LPWNs will be investigated according to the results proposed in this paper, such as state equivalency, liveness, and reachability. The LPWN will be applied efficiently to progress mining.

## Figures and Tables

**Algorithm 1:**
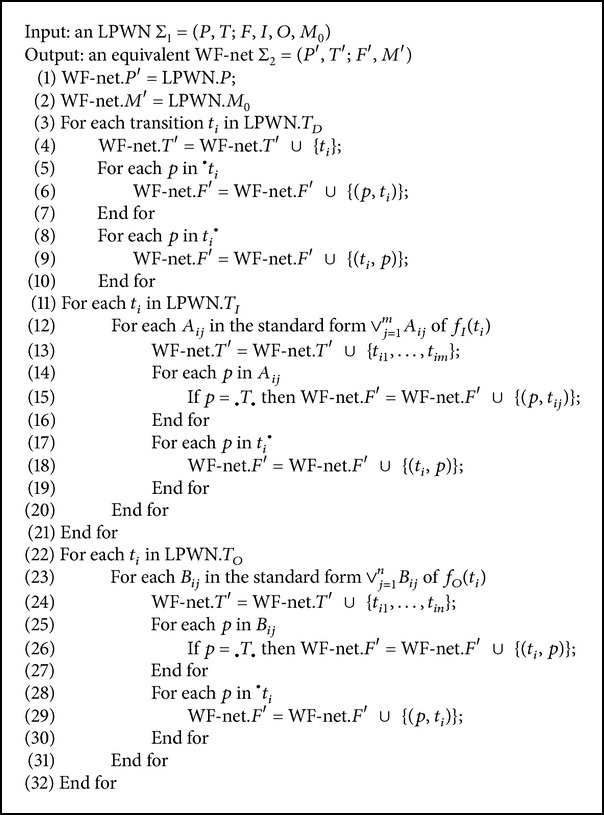
Transforming an LPWN into an equivalent WF-net.

**Table 1:** Logic expressions of all transitions and their standard items.

Transitions	Logic expressions	Standard items		
receive_order	*f*_*I*_(receive_order) = A_11_∨A_12_∨A_13_	A_11_ = *p*_1_∧order_1∧¬order_2	A_12_ = *p*_1_∧order_1∧order_2	A_13_ = *p*_1_∧¬order_1∧order_2
send_to_express	*f*_*O*_(send_to_express) = B_11_∨B_12_∨B_13_	*B*_11_ = *p*_3_∧¬*p*_4_	B_12_ = *p*_3_∧*p*_4_	B_13_ = ¬*p*_3_∧*p*_4_
confirm_goods	*f*_*O*_(confirm_refuses) = B_31_∨B_32_∨B_33_	B_31_ = *p*_6_∧good_1∧¬good_2	B_32_ = *p*_6_∧good_1∧good_2	B_33_ = *p*_5_∧¬good_1∧good_2
confirm_refuses	*f*_*O*_(confirm_goods) = B_21_∨B_22_∨B_23_	B_21_ = *p*_5_∧refuse_1∧¬refuse_2	B_22_ = *p*_5_∧refuse_1∧refuse_2	B_23_ = *p*_5_∧¬refuse_1∧refuse_2
send_money	*f*_*O*_(send_money) = B_41_∨B_42_∨B_43_	B_41_ = *p*_7_∧money_1∧¬money_2	B_42_ = *p*_7_∧money_1∧money_2	B_43_ = *p*_7_∧¬money_1∧money_2
receive_payment	*f*_*I*_(receive_payment) = A_21_∨A_22_∨A_23_	*A*_21_ = *p*_7_∧pay_1∧¬pay_2	A_22_ = *p*_7_∧pay_1∧pay_2	A_12_ = *p*_7_∧¬pay_1∧pay_2

## References

[1] Li Z., Zhou M. (2008). Control of elementary and dependent siphons in Petri nets and their application. *IEEE Transactions on Systems, Man, and Cybernetics, Part A: Systems and Humans*.

[2] Huang Y.-S., Chung T.-H. (2011). Modelling and analysis of air traffic control systems using hierarchical timed coloured Petri nets. *Transactions of the Institute of Measurement and Control*.

[3] Hu H., Zhou M. C., Li Z. (2010). Low-cost and high-performance supervision in ratio-enforced automated manufacturing systems using timed Petri nets. *IEEE Transactions on Automation Science and Engineering*.

[4] Chen W.-L., Kan C.-D., Lin C.-H., Chen T. (2014). A rule-based decision-making diagnosis system to evaluate arteriovenous shunt stenosis for hemodialysis treatment of patients using fuzzy petri nets. *IEEE Journal of Biomedical and Health Informatics*.

[5] Wang Y., Lin C., Ungsunan P. D., Huang X. (2011). Modeling and survivability analysis of service composition using Stochastic Petri Nets. *The Journal of Supercomputing*.

[6] Du Y., Jiang C., Zhou M. (2009). A Petri net-based model for verification of obligations and accountability in cooperative systems. *IEEE Transactions on Systems, Man, and Cybernetics Part A: Systems and Humans*.

[7] Liu W., Du Y., Yan C. (2012). Soundness preservation in composed logical time workflow nets. *Enterprise Information Systems*.

[8] Du Y. Y., Ning Y. H., Qi L. (2014). Reachability analysis of logic Petri nets using incidence matrix. *Enterprise Information Systems*.

[9] de Medeiros A. K. A., Weijters A. J. M. M., van der Aalst W. M. P. (2007). Genetic process mining: an experimental evaluation. *Data Mining and Knowledge Discovery*.

[10] van der Aalst W. M. P. (2011). *Process Mining: Discovery, Conformance and Enhancement of Business Processes*.

[11] Tan W., Zhou M. (2013). *Business and Scientific Workflows: A Service-Oriented Approach*.

[12] Yu W., Yan C., Ding Z., Jiang C., Zhou M. (2014). Modeling and validating E-commerce business process based on petri
nets. *IEEE Transactions on Systems, Man, and Cybernetics: Systems*.

[13] Dijkman R. M., Dumas M., Ouyang C. (2008). Semantics and analysis of business process models in BPMN. *Information and Software Technology*.

[14] van der Aalst W. M. P., van Hee K. M., ter Hofstede A. H. M. (2011). Soundness of workflow nets: classification, decidability, and analysis. *Formal Aspects of Computing*.

[15] van der Aalst W. M. P., Rubin V., Verbeek H. M. W., van Dongen B. F., Kindler E., Günther C. W. (2010). Process mining: a two-step approach to balance between underfitting and overfitting. *Software & Systems Modeling*.

[16] Ingvaldsen J. E., Gulla J. A. (2012). Industrial application of semantic process mining. *Enterprise Information Systems*.

[17] Wil van der Aalst M. P. (2013). Decomposing Petri nets for process mining: a generic approach. *Distributed and Parallel Databases*.

[18] Goeminne M., Mens T. (2013). A comparison of identity merge algorithms for software repositories. *Science of Computer Programming*.

[19] Chen Y., Alspaugh S., Katz R. (2012). Interactive analytical processing in big data systems: a cross-industry study of MapReduce workloads. *Proceedings of the VLDB Endowment*.

